# Serum microRNAs as non-invasive biomarkers for cancer

**DOI:** 10.1186/1476-4598-9-306

**Published:** 2010-11-26

**Authors:** Jan C Brase, Daniela Wuttig, Ruprecht Kuner, Holger Sültmann

**Affiliations:** 1 Working Group Cancer Genome Research, German Cancer Research Center, Heidelberg, Germany

## Abstract

Human serum and other body fluids are rich resources for the identification of novel biomarkers, which can be measured in routine clinical diagnosis. microRNAs are small non-coding RNA molecules, which have an important function in regulating RNA stability and gene expression. The deregulation of microRNAs has been linked to cancer development and tumor progression. Recently, it has been reported that serum and other body fluids contain sufficiently stable microRNA signatures. Thus, the profiles of circulating microRNAs have been explored in a variety of studies aiming at the identification of novel non-invasive biomarkers.

In this review, we discuss recent findings indicating that circulating microRNAs are useful as non-invasive biomarkers for different tumor types. Additionally, we summarize the knowledge about the mechanism of microRNA release and the putative functional roles of circulating microRNAs. Although several challenges remain to be addressed, circulating microRNAs have the potential to be useful for the diagnosis and prognosis of cancer diseases.

## Background

One of the major challenges in cancer research is the identification of stable biomarkers, which can be routinely measured in easily accessible samples. Over many decades it has been shown that cell-free DNA and RNA is present in serum and other body fluids and that these circulating nucleic acids may represent potential biomarkers (reviewed in [1-3]). One decade ago, microRNAs (miRNAs) were discovered as a novel class of evolutionarily conserved small (18 - 24 nucleotides) non-coding RNA molecules, which are important regulators of gene expression [4,5]. By targeting the 3' untranslated region (UTR) of mRNA transcripts, miRNAs influence RNA stability and translational efficiency via degradation or protein translation inhibition, respectively [4,6-9]. Thus, alterations in miRNA-expression can affect crucial biological processes in cancer development and progression, such as proliferation, differentiation and apoptosis [10,11]. For example, the well-described *let 7 *miRNA family is evolutionary conserved from *C. elegans *to humans and has been shown to directly target the human *RAS *oncogenes [12]. In humans, significantly reduced expression of let-7 family members has been found in various cancers including lung, colon, ovarian, and gastric cancer, as well as leiomyoma and melanoma [13]. Its decreased expression is associated with a significantly shorter survival of lung cancer patients and promotes the colony forming ability of lung cancer cells *in vitro *[14]. *miRNA 221 *and *miRNA 222 *are located on chromosome X, and have been reported to be up regulated in various tumor types such as ovarian cancer [15], hepatocellular cancer [16] and glioblastomas [17]. Both miRNAs were shown to promote cell growth, cell cycle progression and invasion in these cancer types *in vitro *and *in vivo *[16,17]. Thus, they act as so-called "oncomirs". These effects are mediated by the direct inhibition of the tumor suppressors *PTEN *[18] and *CDKN1B *[15].

Similar to mRNA, the expression of miRNAs can be influenced by several factors, e.g. chromosomal re-arrangements, promoter methylation changes and regulation of transcription, all of which are known to occur in human malignancies [19-22].

## Cell-free miRNAs and possible release mechanisms

While miRNA presence is relevant for the regulation of cancer-associated genes in tissues, the possibility to extract and reliably determine cell-free miRNA content in body fluids like serum was first shown in 2008 [23]. This finding was confirmed by a subsequent study revealing that miRNAs are enriched in the small RNA fraction isolated from serum samples [24]. Cell-free miRNAs in body fluids are stable under harsh conditions including boiling, low/high pH, extended storage and multiple freeze-thaw cycles [24-28]. In contrast, synthetic miRNAs were found to be quickly degraded by the high levels of RNAse activity in plasma [25]. Filtering and differential centrifugation experiments suggest that miRNAs are not derived from cells circulating in the blood [25]. At present, there are at least two possible explanations for the stability and origin of circulating miRNAs:

One hypothesis is that passive release occurs during tissue injury. For example, *miRNA-208 *was shown to be exclusively expressed in the heart and was measured in the serum after heart tissue injury [29]. The same unspecific release could also exist in cancer, since the high rate of proliferation and cell lysis in tumors might contribute to the abundance of miRNAs in the blood stream. Alternatively, miRNAs are contained in small particles and are therefore protected against RNase activity. Recently, it has been shown that a transfer of mRNA and miRNA between cells can be accomplished through microvesicles [30]. These are small (50 nm to 100 nm) particles, which are shed from the cell plasma membrane into the extracellular space and released into the blood stream [31,32]. Microvesicles are derived from different cell types, e.g. reticulocytes, dendritic cells, B/T cells and mast cells [33-37]. Additionally, it was shown that non-hematopoietic cells like intestinal epithelial cells and neuroglial cells are capable to release microvesicles [38,39].

## Putative functional roles of circulating miRNAs

Microvesicles, also known as exosomes, are supposed to be important for cell-cell communication. However, the role of incorporated miRNA molecules is unclear. Hunter et al. compared the expression levels of miRNAs from microvesicles with that of platelets and peripheral blood mononuclear cells in healthy individuals [40]. Significant differences were found and target prediction demonstrated that the majority of the miRNAs from the microvesicles are involved in the regulation of hematopoiesis and cellular differentiation [40].

Recently, the mechanism of microvesicle based miRNA release was reported to involve a ceramide-dependent secretory machinery [41]: Kosaka and colleagues showed that miRNA abundance changes after overexpression and inhibition of a rate-limiting enzyme involved in ceramide biosynthesis [41]. Interestingly, they also found that miRNAs are transported in microvesicles and exert gene silencing in recipient cells. The recent data suggest that cells can actively secrete endogenous miRNAs. However, it remains unclear whether there is a specific mechanism leading to an increase of certain selected miRNAs.

Although the exact mechanism for microvesicle formation and nucleic acid incorporation is still unknown, exosomes seem to have important roles in cell-cell communication [32]. Therefore, the contained miRNAs could also have important functions in tumor development and progression: In 1979, it was shown that tumor related exosomes are present in the blood of women suffering from ovarian cancer [42]. Recently, the quantity of tumor-derived exosomes in the peripheral circulation has been found to be highly correlated with ovarian cancer stages [26]. Moreover, the miRNA content of tumor cell-derived exosomes is correlated to the miRNA level in the primary tumor [26,43]. Skog et al. reported that glioblastoma-derived RNA contained in microvesicles is functional and is taken up by and processed in human brain microvascular endothelial cells (HBMVEC) in cultures [44]. This lead to the hypothesis that tumor cells use exosomes to transport genetic information, including miRNAs, to surrounding cells and thereby support tumor growth and progression [44]. If this hypothesis holds true, miRNAs could be suitable candidates to manipulate the microvesicles' target cells by regulating their RNA stability and translation. Moreover, circulating miRNAs might modulate immune responses [45,46]: For example, microvesicles derived from human melanoma and colon cancer can promote tumor growth and immune escape by mediating the differentiation of monocytes towards TGFβ-secreting myeloid suppressive cells [45]. However, it has not been investigated if these effects are mediated by the miRNAs contained in the microvesicles.

## Circulating miRNAs reflect physiological and pathological changes

In healthy individuals, the levels of cell-free miRNAs present in sera are stable [24,27]. Under healthy conditions, the serum miRNA profile is similar to that of circulating blood cells [24]. Thus, alterations of serum miRNA levels may be indicative of physiological or pathological changes and may possibly be used as surrogate biomarkers [47]. For example, circulating miRNAs were found in the sera of pregnant women [27]: *miRNA-526a*, *miRNA-527 *and *miRNA-520d-5p *showed a considerably high fold-change and could be used to distinguish pregnant from non-pregnant women with high accuracy. Moreover, placenta derived miRNAs (e.g. *miRNA 141*, *miRNA 149*, *miRNA 299 5p *and *miRNA 517a*) are detectable in the maternal plasma, and their concentrations decrease directly after childbirth [48,49]. Therefore, miRNAs have been discussed as novel non-invasive markers for prenatal diagnosis [48].

As to pathological changes, tissue specific miRNAs were analyzed in the blood stream as markers for myocardial injury and drug induced liver injury: A rat model of acute myocardial infarction demonstrated that the plasma levels of the cardiac-specific *miRNA-208 *and *miRNA-499 *are increased in this disease [50]. These miRNAs are also elevated in the plasma of human patients with acute myocardial infarction [50,51]. Drug induced liver injury, a frequent side effect which significantly influences the patient's health and treatment costs, is associated with increased plasma levels of *miRNA 122 *and *miRNA 192 *in a mouse model [52].

Cell-free miRNAs are also associated with inflammatory diseases [53,54]: Circulating *miRNA-146a *and *miRNA-223 *were significantly reduced in septic patients when compared to patients with a systemic inflammatory response syndrome or healthy controls [53]. Furthermore, reduced plasma levels of *miRNA 132 *were observed in patients with rheumatoid arthritis and osteoarthrosis compared to healthy controls [55].

## Serum miRNAs as biomarkers for cancer diagnosis

The availability of novel biomarkers could improve diagnosis and the clinical management of cancer. A perfect biomarker should be easily accessible in a noninvasive manner. Therefore, miRNA profiles in serum and plasma samples from cancer patients have been screened to identify novel biomarkers for the diagnosis of tumors (summarized in Table 1):

**Table 1:** Circulating miRNAs in the serum as diagnostic markers for different tumor entities

Tumor entity	References	Study Design	Sample Size	Circulating miRNAs examined	Technology	Normalization	Promising circulating miRNAs
**B-Cell Lymphoma**	Lawrie et al. [23]	Tumor vs. normal, retrospective study on prognosis	60 patients vs. 43 healthy controls	3	Quantitative RT-PCR	*miRNA-16*	*miRNA-155, miRNA-210*, and *miRNA-21*

**Breast Cancer**	Heneghan et al. [73]	Tumor vs. normal	83 patients vs. 44 healthy controls	7	Quantitative RT-PCR	*miRNA-16*	*miRNA-195 *and *let7a*
	
	Zhu et al. [83]	Tumor vs. normal	13 patients vs. 8 healthy controls	3	Quantitative RT-PCR	18 s rRNA	*miRNA-155*

**Colon Cancer**	Huang et al. [60]	Tumor vs. normal	Screening: 20 patients vs. 20 healthy controls Validation: 80 patients, 37 adenomas and 39 healthy controls	12	Quantitative RT-PCR	*miRNA-16*	*miRNA-29 *and *miRNA92a*
	
	Ng et al. [57]	Tumor vs. normal, tissue and serum	Screening: 5 plasma samples, associated tumor/normal tissue 1.validation: 25 patients vs. 20 healthy controls 2. validation 180 samples	95	Quantitative RT-PCR Array	*RNU6B*	*miR-17-3p *and *miR-92*

**Gastric Cancer**	Tsujiura et al. [85]	Tumor vs. Normal	Screening: 8 samples and associated tissue Validation: 69 patients vs. 30 healthy controls	5	Quantitative RT-PCR	*RNU6B*	*miR-17-5p, miR-21*, *miR-106a, miR-106b *and *let-7a*

**Leukemia**	Tanaka et al. [56]	Tumor vs. Normal	Screening: 2 patients vs. 7 healthy controls Validation: 61 patients vs. 16 healthy controls	723	microRNA Microarray (Agilent Technologies)	*miRNA-638*	*miRNA-92a*

**Lung Cancer**	Chen et al. [24]	Tumor vs. normal	Screening: Pool analysis Validation: 152 patients vs. 75 healthy controls	Genome-wide profiling by Solexa sequencing	Solexa sequencing, Quantitative RT-PCR	Directly normalized to total RNA	*miRNA-25 *and *miRNA-223*
	
	Hu et al. [74]	Study on prognosis (Overall survival)	Screening: 60 patients Validation: 243 patients	Genome-wide profiling by Solexa sequencing	Solexa sequencing, Quantitative RT-PCR	Referenced to control healthy serum sample	*miR-486, miR-30 d, miR-1 *and *miR- 499*

**Oral Cancer**	Liu et al. [80]	Tumor vs. normal	43 patients vs. 21 healthy controls	1	Quantitative RT-PCR arrays	*miRNA-16*	*miR-31*

**Ovarian Cancer**	Resnick et al. [67]	Tumor vs. normal	Screening: 9 patients vs. 4 healthy controls Validation: 19 patients vs. 11 healthy controls	365	Quantitative RT-PCR arrays	*U44/U48 and miRNA-142-3p*	*miRNA-21, miRNA-92, miRNA-93, miRNA-126, miRNA-29a, miRNA-155, miRNA-127 *and *miRNA-99b*

**Pancreatic Cancer**	Ho et al. [28]	Tumor vs. normal	Screening: 11 patients vs. 14 healthy controls, Validation: 11 patients vs. 11 healthy controls	1	Quantitative RT-PCR arrays	c. elegans spike-in *miRNA-54*	*miRNA-210*
	
	Wang et al. [61]	Tumor vs. normal	49 patients vs. 36 healthy controls	4	Quantitative RT-PCR arrays	*miRNA-16*	*miR-21, miR-210, miR-155*, and *miR-196a*

**Prostate Cancer**	Mitchell et al. [25]	Tumor vs. normal	Screening: Pool analysis Validation: 25 patients vs. 25 healthy controls	6	Quantitative RT-PCR	c. elegans spike-in *cel-miR-39*, *celmiR-54*, and *cel-miR-238*	*miRNA-141*
	
	Brase et al. [72]	Low grade vs. high grade	Screening: 7 high grade vs. 14 low grade Validation: 116 patients	667	Quantitative RT-PCR arrays	c. elegans spike-in *cel-miR-39*, *celmiR-54*, and *cel-miR-238*	*miRNA-141*, *miRNA-375*

**Squamous Cell Carcinoma**	Wong et al. [81]	Tumor vs. Normal tissue screening, Validation in serum	30 patients vs. 38 healthy controls	1	Quantitative RT-PCR arrays	*miRNA-16*	*miRNA-184*

Lawrie et al. were the first to discover tumor specific deregulation of circulating miRNAs. Their study demonstrated that *miRNA-21 *is highly abundant in the sera of diffuse large B-cell lymphoma patients [23]. miRNA profiling of leukemia patients has shown circulating *miRNA-92a *to be considerably downregulated in the case of malignancy [56]. In 2008, Mitchell et al. reported that miRNAs derived from epithelial tumors are also rapidly released into the blood stream [25]. They used a mouse model to show that human miRNAs can be detected in the blood of mice after prostate cancer xenograft transplantation. The amount of human miRNAs was correlated with the xenograft tumor mass. These results clearly demonstrated that tumor-derived miRNAs can enter the circulation even when originating from epithelial cancers. Additionally, Mitchell et al. found circulating *miRNA-141 *to be significantly elevated in sera of metastatic prostate cancer when compared to those of healthy controls [25]. Ng and colleagues employed quantitative polymerase chain reaction (qPCR) to analyze miRNA profiles in colon cancer cells, the corresponding adjacent normal colonic tissue and plasma of patients and healthy controls [57,58]: In this study, five miRNAs were found to be significantly overexpressed in the tumor cells as well as more abundant in the plasma samples of tumor patients compared to those of healthy volunteers (Table 1). *miRNA-92 *and *miRNA-17-3p *were confirmed as diagnostic markers for colon cancer in an independent validation study [57]. Both miRNAs belong to the *miRNA-17-92 *gene cluster, which is supposed to be involved in cancer pathogenesis [59].

Circulating miRNAs were analyzed for the feasibility to detect early cancer development. Huang et al. studied the miRNA profiles in the blood stream of early stage colon cancer patients. Interestingly, they identified circulating miRNAs which distinguished adenomas from healthy controls with a 73% sensitivity and a 79% specificity [60]. These data indicate that cell-free miRNAs are promising markers for early tumor diagnosis. Novel biomarkers could be particularly useful for an earlier detection of tumors, which are clinically asymptomatic for extended periods. Pancreatic cancer, for instance, is often diagnosed in an advanced stage and associated with very short survival times. *miRNA-21*, *miRNA-155*, *miRNA-196a *and *miRNA-210*, which are known to be associated with pancreatic cancer, were also found to be elevated in the plasma of pancreatic carcinoma patients [28,61]. These miRNAs have been largely studied for their functional consequences: *miRNA-21 *is known to be upregulated in several tumor entities and targets known tumor suppressive genes like *PTEN *and *PDCD4 *[62,63]. *miRNA-155 *has been found to downregulate the proapoptotic protein *TP53INP1 *[64]. *miRNA-196a *was described to be a prognostic marker in pancreatic cancer [65]. *miRNA-210 *is directly regulated by HIF1A and therefore induced by hypoxia [66].

The performance of circulating miRNAs as diagnostic markers has been compared to established blood-based markers: Women with high risk for ovarian cancer can undergo a CA 125 serum-based screening test. However, with a sensitivity of 40%, CA-125 is a poor marker for the early detection of this tumor. A set of serum miRNAs has recently been found to be highly abundant in patients with ovarian cancer. Some of these miRNAs were also deregulated in patients exhibiting normal CA-125 serum levels [67]. These data indicate that circulating miRNAs might be non-invasive markers, which could contribute to improving established clinical diagnostic tests.

Serum miRNA profiles among different cancer types have also been analyzed: Chen and colleagues compared the miRNA levels between colorectal and lung cancer patients [24]. Several miRNAs (e.g., *miR-134*, *miR-146a*, *miR-221*, *miR-222*, *miR-23a*) were significantly deregulated in the sera of both patient groups, suggesting that a miRNA profile common for several types of cancers may exist. On the other hand, tumor-specific miRNAs were also described: Lodes et al. analyzed the serum miRNA profiles in five tumor entities (prostate, colon, ovarian, breast and lung cancer) using a high-density microarray. Here, different tumor entities could be distinguished based on the miRNA profiles in patients sera [68].

## Circulating miRNAs are correlated with tumor progression

The findings discussed in the previous paragraph were based on comparisons of the miRNA serum levels between tumor patients and healthy controls. None of the described circulating miRNAs for the diagnosis of epithelial tumors showed correlations to histological subtypes, different stages or grades of cancer in further validation studies [56,57,61,67]. This suggests that the identified circulating miRNAs might be promising biomarkers for cancer detection but not necessarily appropriate for the prediction of the clinical courses of the diseases. In contrast, in tissue samples, miRNAs seem to be valuable markers to predict the clinical outcomes of cancer patients [69-71].

In our own study [72], we analyzed the miRNA profiles in sera of patients with highly aggressive compared to localized prostate cancer. Several circulating miRNAs were considerably higher abundant in patients with metastatic disease. Two independent validation studies indicated that *miRNA-141 *and *miRNA-375 *were the most promising markers correlated with prostate tumor progression. Recently, circulating miRNAs have also been reported to be correlated with clinicopathological variables (nodal and estrogen receptor status) in patients with breast cancer [73]. However, this study as well as our own data only demonstrated that circulating miRNAs are correlated with histopathological parameters, and not directly associated with patients' outcome. To evaluate the prognostic potential of the identified circulating miRNAs, larger retrospective validation studies integrating long-term follow-up data are required.

For lung cancer, it was shown that serum miRNAs are promising prognostic biomarkers: Hu et al. demonstrated that circulating miRNAs can be used to predict the clinical outcomes of non-small-cell lung cancer (NSCLC) patients [74]. In their screening study, the authors compared the serum miRNA profiles of patients with long and short survival times using Solexa sequencing and validated the abundance of 13 selected miRNAs in 243 patients by qPCR. Four miRNAs (*miRNA-486*, *miRNA-30d*, *miRNA-1*, and *miRNA-499*) were confirmed to be associated with patient outcome. *miRNA-1 *was already described to be significantly downregulated in lung cancer tissue, thereby leading to decreased cell proliferation, migration and motility. *miRNA-1 *is supposed to target the *MET *oncogene as well as the gene *HDAC4 *[75]. Hu et al. also demonstrated that a combination of selected circulating miRNAs had a higher sensitivity than single biomarkers: Patients exhibiting large amounts of two or more high-risk miRNAs in the serum had a significantly increased probability of shorter survival times. Taken together, these results indicate a significant association of circulating miRNAs with the survival of NSCLC patients and therefore suggest that miRNAs are useful as prognostic markers.

## Technical advances and challenges to analyze circulating miRNA

Due to the small amount of circulating miRNAs and the large amount of proteins, miRNA extraction from serum samples is technically challenging. To this end, several phenol/chloroform-based extraction protocols are available. Commercially available extraction kits without acid phase separation can also be used for the isolation of miRNA from body fluids.

Circulating miRNAs can be extracted from both serum as well as plasma samples. Serum has recently been described to yield lower amounts of circulating miRNAs compared to plasma [76]. In addition, the usefulness of serum samples has been questioned since the range of miRNAs from different samples can vary [76]. However, due to practical reasons, in the clinical routine, mainly serum samples are available. To this end, a good correlation was observed when the individual miRNA levels were compared between serum and plasma samples from the same patient donors [25]. Thus, both sample types seem to be suitable for the analysis of cell-free miRNAs.

One of the main problems associated with circulating miRNA extraction and comparison of sample collectives is the quantification of the miRNA. The low abundance of miRNA in serum can hardly be determined using spectrophotometers. A robust and sensitive method for the analysis of serum miRNAs is their relative quantification by a stem-loop reverse transcription PCR (RT-PCR), which has been widely used for the sensitive detection of low abundant circulating miRNAs [77] with high reproducibility. New technologies for serum-based miRNA analysis are emerging: For example, Lusi et al. designed a PCR- and label-free, sensitive detection method [78] based on an electrochemical sensor. After the hybrid formation of the miRNA with an inosine substitute, the oxidation of guanine generates an electrical signal, which can be quantified [78]. Microarray-based expression analysis is challenging since a large amount of RNA is needed for the analyses. Lodes et al. reported similar limits of detection for the analysis of circulating miRNAs for microarrays when compared to qPCR-based methods [68]. Deep sequencing technologies have resulted in a steep increase of the rate of newly described microRNAs [79]. Since 2007, almost all newly recovered microRNAs were derived from deep sequencing analyses. The current release (miRBase 16) encompasses over 15.000 microRNA gene loci. The user can search for tissue- and stage specific expression, and compare own data with microRNA profiles in different diseases. First studies indicate disease-specific fingerprints in serum [24,74]. Thus, large-scale miRNA sequencing appears to be very promising with respect to the identification of further biomarkers.

Due to the described technical and quality variations, the strategy of raw data normalization is a critical issue. Since endogenous ("housekeeping") controls do not exist, variances based on starting material and miRNA extraction have to be carefully balanced. So far, several normalization strategies for the analysis of circulating miRNAs are available (Table 1). Literature-based tissue housekeeping genes or miRNAs in the serum have been used for data normalization. For example, *miRNA-16 *was used as a reference for serum miRNA analysis in several studies [23,60,61,73,80,81] (Table 1). This miRNA is consistently expressed in different human tissue entities [82] and is also detectable in serum samples. However, measurements of *miRNA-16 *in sera seem to be inconsistent. While some studies demonstrated a lack of significant differences between clinically defined entities [61,73], *miRNA-16 *was also found to be highly abundant in the sera of prostate cancer patients [68]. Small non-coding RNAs are also commonly used. However, these have been reported to show limitations due to degradation in the blood stream [23,60,83]. Mitchell and colleagues reported a spike-in normalization approach to control for technical variances during the purification process. Three *C. elegans *miRNAs (without sequence similarity to known human miRNAs) were included in the purification procedure and used for data normalization [25,84].

The mentioned challenges concerning extraction, quantification and data processing steps in miRNA analysis may lead to considerably variable results and clearly demonstrate the need for better standardization methods.

## Future directions

Circulating miRNAs offer great hope for the diagnosis and prognosis, and possibly prediction, of cancer. However, there are still limitations to the technology and the recent study designs. In-line with the lessons learned from gene expression profiling, clinical associations of miRNA presence identified in small sample cohorts have to be verified in larger and independent studies, and the efforts to translate the findings into clinical routine have to be increased. Initial data of miRNA in several tumor entities were either based on literature search or on a limited number of miRNAs (Table 1). Global screening studies monitoring a large set of human miRNAs are likely to lead to the discovery of better markers for specific diseases. In terms of biological or cellular functions, there is less known for any of the discovered serum miRNA. There are still controversial results concerning the relation of miRNA levels between tissue and corresponding serum: Several researchers suggested that tumor-associated miRNAs should be evaluated in the serum *and *in the tumor tissue [25,57]. This is conceptually in line with the role of microvesicles in neoplastic progression [44]. However, in lung cancer, the let-7 family was shown to be associated with clinical outcome in tissue samples only, and was not detected in the serum [74]. Thus, circulating miRNAs may not always be directly associated with the changes occurring in tumor tissues but may also reflect indirect effects. On the other hand, deregulated circulating miRNAs have been reported to be significantly reduced in post-operative states [60,73,80,81,85].

Wang et al. concluded from their study that specific miRNAs are released into the blood stream after drug induced liver injury leading to a downregulation in the tissue [52]. In contrast to that, Tanaka et al. speculated that tumor cells rely on the specific intake of miRNAs from circulating microvesicles [56]. These contradictive assumptions clearly demonstrate a need for additional studies to elucidate the relation and function of tissue and serum miRNA expression levels. Furthermore, additional studies focusing on tumor specific microvesicles may provide insights into the biological roles of circulating miRNAs.

Finally, it is unknown how soon miRNA changes appear in the serum, although some first results showed that miRNAs occur early in the blood stream during colon cancer development [60] and after drug induced liver injury [52]. Serum miRNAs-122 and 192 are detectable prior to the routine detection of liver injury using an alanine aminotransferase enzyme test [52]. So far, no study has analyzed the influence of age, health conditions or dynamical changes of the serum miRNA profile in different individuals. Therefore, the kinetics of circulating miRNAs should be analyzed in detail to unravel if infection diseases or lifestyle changes can lead to changes in the serum and to correct for these changes in future studies. Additionally it is unknown, if medical treatment leads to a change of the serum miRNA profiles. *In-vitro *studies revealed that specific microRNAs impact on drug sensitivity [86]. Thus, it is possible that personal treatments are reflected by serum microRNA profiles. This aspect is highly relevant, since the individual treatment may influence the value of novel non-invasive miRNA biomarkers.

## Conclusions

Although we are just beginning to understand the role and function of circulating miRNAs in the serum, miRNAs in body fluids are currently extensively explored for their potential as non-invasive diagnostic tumor markers. Tumor-specific circulating miRNAs may improve cancer diagnosis and prognosis, since several promising miRNAs have already been described as non-invasive biomarkers for different tumor entities. However, larger sample sets including long-term clinical data are urgently required for future studies. In contrast, circulating miRNAs for the prediction of drug responses have not been described so far. Isolation, quantification and normalization strategies have to be standardized before any of the novel miRNA biomarkers is applicable for clinical routine.

## Competing interests

The authors declare that they have no competing interests.

## Authors' contributions

JCB reviewed the literature, wrote and drafted the manuscript; RK, DW and HS corrected and finalized the manuscript. All authors read and approved the final version.

## References

[B1] Swarup V Rajeswari MR Circulating (cell-free) nucleic acids--a promising, non-invasive tool for early detection of several human diseases FEBS Lett 2007 581 795 799 10.1016/j.febslet.2007.01.051 17289032

[B2] Fleischhacker M Schmidt B Circulating nucleic acids (CNAs) and cancer--a survey Biochim Biophys Acta 2007 1775 181 232 17137717 10.1016/j.bbcan.2006.10.001

[B3] Tsang JC Lo YM Circulating nucleic acids in plasma/serum Pathology 2007 39 197 207 10.1080/00313020701230831 17454749

[B4] Bartel DP MicroRNAs: genomics, biogenesis, mechanism, and function Cell 2004 116 281 297 10.1016/S0092-8674(04)00045-5 14744438

[B5] Ambros V The functions of animal microRNAs Nature 2004 431 350 355 10.1038/nature02871 15372042

[B6] Bushati N Cohen SM microRNA functions Annu Rev Cell Dev Biol 2007 23 175 205 10.1146/annurev.cellbio.23.090506.123406 17506695

[B7] Yekta S Shih IH Bartel DP MicroRNA-directed cleavage of HOXB8 mRNA Science 2004 304 594 596 10.1126/science.1097434 15105502

[B8] He L Hannon GJ MicroRNAs: small RNAs with a big role in gene regulation Nat Rev Genet 2004 5 522 531 10.1038/nrg1379 15211354

[B9] Ambros V microRNAs: tiny regulators with great potential Cell 2001 107 823 826 10.1016/S0092-8674(01)00616-X 11779458

[B10] Miska EA How microRNAs control cell division, differentiation and death Curr Opin Genet Dev 2005 15 563 568 10.1016/j.gde.2005.08.005 16099643

[B11] Esquela-Kerscher A Slack FJ Oncomirs - microRNAs with a role in cancer Nat Rev Cancer 2006 6 259 269 10.1038/nrc1840 16557279

[B12] Johnson SM Grosshans H Shingara J Byrom M Jarvis R Cheng A Labourier E Reinert KL Brown D Slack FJ RAS is regulated by the let-7 microRNA family Cell 2005 120 635 647 10.1016/j.cell.2005.01.014 15766527

[B13] Peter ME Let-7 and miR-200 microRNAs: guardians against pluripotency and cancer progression Cell Cycle 2009 8 843 852 19221491 10.4161/cc.8.6.7907 PMC2688687

[B14] Takamizawa J Konishi H Yanagisawa K Tomida S Osada H Endoh H Harano T Yatabe Y Nagino M Nimura Y Reduced expression of the let-7 microRNAs in human lung cancers in association with shortened postoperative survival Cancer Res 2004 64 3753 3756 10.1158/0008-5472.CAN-04-0637 15172979

[B15] Wurz K Garcia RL Goff BA Mitchell PS Lee JH Tewari M Swisher EM MiR-221 and MiR-222 alterations in sporadic ovarian carcinoma: Relationship to CDKN1B, CDKNIC and overall survival Genes Chromosomes Cancer 2010 49 577 584 20461750 10.1002/gcc.20768 PMC2869465

[B16] Pineau P Volinia S McJunkin K Marchio A Battiston C Terris B Mazzaferro V Lowe SW Croce CM Dejean A miR-221 overexpression contributes to liver tumorigenesis Proc Natl Acad Sci USA 2010 107 264 269 10.1073/pnas.0907904107 20018759 PMC2806773

[B17] Zhang J Han L Ge Y Zhou X Zhang A Zhang C Zhong Y You Y Pu P Kang C miR-221/222 promote malignant progression of glioma through activation of the Akt pathway Int J Oncol 2010 36 913 920 20198336 10.3892/ijo_00000570

[B18] Chun-Zhi Z Lei H An-Ling Z Yan-Chao F Xiao Y Guang-Xiu W Zhi-Fan J Pei-Yu P Qing-Yu Z Chun-Sheng K MicroRNA-221 and microRNA-222 regulate gastric carcinoma cell proliferation and radioresistance by targeting PTEN BMC Cancer 2010 10 367 10.1186/1471-2407-10-367 20618998 PMC2914702

[B19] Calin GA Croce CM MicroRNA signatures in human cancers Nat Rev Cancer 2006 6 857 866 10.1038/nrc1997 17060945

[B20] Rosenfeld N Aharonov R Meiri E Rosenwald S Spector Y Zepeniuk M Benjamin H Shabes N Tabak S Levy A MicroRNAs accurately identify cancer tissue origin Nat Biotechnol 2008 26 462 469 10.1038/nbt1392 18362881

[B21] Chen CZ MicroRNAs as oncogenes and tumor suppressors N Engl J Med 2005 353 1768 1771 10.1056/NEJMp058190 16251533

[B22] Caldas C Brenton JD Sizing up miRNAs as cancer genes Nat Med 2005 11 712 714 10.1038/nm0705-712 16015356

[B23] Lawrie CH Gal S Dunlop HM Pushkaran B Liggins AP Pulford K Banham AH Pezzella F Boultwood J Wainscoat JS Detection of elevated levels of tumour-associated microRNAs in serum of patients with diffuse large B-cell lymphoma Br J Haematol 2008 141 672 675 10.1111/j.1365-2141.2008.07077.x 18318758

[B24] Chen X Ba Y Ma L Cai X Yin Y Wang K Guo J Zhang Y Chen J Guo X Characterization of microRNAs in serum: a novel class of biomarkers for diagnosis of cancer and other diseases Cell Res 2008 18 997 1006 10.1038/cr.2008.282 18766170

[B25] Mitchell PS Parkin RK Kroh EM Fritz BR Wyman SK Pogosova-Agadjanyan EL Peterson A Noteboom J O'Briant KC Allen A Circulating microRNAs as stable blood-based markers for cancer detection Proc Natl Acad Sci USA 2008 105 10513 10518 10.1073/pnas.0804549105 18663219 PMC2492472

[B26] Taylor DD Gercel-Taylor C MicroRNA signatures of tumor-derived exosomes as diagnostic biomarkers of ovarian cancer Gynecol Oncol 2008 110 13 21 10.1016/j.ygyno.2008.04.033 18589210

[B27] Gilad S Meiri E Yogev Y Benjamin S Lebanony D Yerushalmi N Benjamin H Kushnir M Cholakh H Melamed N Serum microRNAs are promising novel biomarkers PLoS ONE 2008 3 e3148 10.1371/journal.pone.0003148 18773077 PMC2519789

[B28] Ho AS Huang X Cao H Christman-Skieller C Bennewith K Le QT Koong AC Circulating miR-210 as a Novel Hypoxia Marker in Pancreatic Cancer Transl Oncol 2010 3 109 113 20360935 10.1593/tlo.09256 PMC2847318

[B29] Ji X Takahashi R Hiura Y Hirokawa G Fukushima Y Iwai N Plasma miR-208 as a biomarker of myocardial injury Clin Chem 2009 55 1944 1949 10.1373/clinchem.2009.125310 19696117

[B30] Valadi H Ekstrom K Bossios A Sjostrand M Lee JJ Lotvall JO Exosome-mediated transfer of mRNAs and microRNAs is a novel mechanism of genetic exchange between cells Nat Cell Biol 2007 9 654 659 10.1038/ncb1596 17486113

[B31] Caby MP Lankar D Vincendeau-Scherrer C Raposo G Bonnerot C Exosomal-like vesicles are present in human blood plasma Int Immunol 2005 17 879 887 10.1093/intimm/dxh267 15908444

[B32] van Niel G Porto-Carreiro I Simoes S Raposo G Exosomes: a common pathway for a specialized function J Biochem 2006 140 13 21 10.1093/jb/mvj128 16877764

[B33] Escola JM Kleijmeer MJ Stoorvogel W Griffith JM Yoshie O Geuze HJ Selective enrichment of tetraspan proteins on the internal vesicles of multivesicular endosomes and on exosomes secreted by human B-lymphocytes J Biol Chem 1998 273 20121 20127 10.1074/jbc.273.32.20121 9685355

[B34] Andre F Schartz NE Movassagh M Flament C Pautier P Morice P Pomel C Lhomme C Escudier B Le Chevalier T Malignant effusions and immunogenic tumour-derived exosomes Lancet 2002 360 295 305 10.1016/S0140-6736(02)09552-1 12147373

[B35] Valenti R Huber V Filipazzi P Pilla L Sovena G Villa A Corbelli A Fais S Parmiani G Rivoltini L Human tumor-released microvesicles promote the differentiation of myeloid cells with transforming growth factor-beta-mediated suppressive activity on T lymphocytes Cancer Res 2006 66 9290 9298 10.1158/0008-5472.CAN-06-1819 16982774

[B36] Thery C Regnault A Garin J Wolfers J Zitvogel L Ricciardi-Castagnoli P Raposo G Amigorena S Molecular characterization of dendritic cell-derived exosomes. Selective accumulation of the heat shock protein hsc73 J Cell Biol 1999 147 599 610 10.1083/jcb.147.3.599 10545503 PMC2151184

[B37] Raposo G Nijman HW Stoorvogel W Liejendekker R Harding CV Melief CJ Geuze HJ B lymphocytes secrete antigen-presenting vesicles J Exp Med 1996 183 1161 1172 10.1084/jem.183.3.1161 8642258 PMC2192324

[B38] Fevrier B Vilette D Archer F Loew D Faigle W Vidal M Laude H Raposo G Cells release prions in association with exosomes Proc Natl Acad Sci USA 2004 101 9683 9688 10.1073/pnas.0308413101 15210972 PMC470735

[B39] van Niel G Raposo G Candalh C Boussac M Hershberg R Cerf-Bensussan N Heyman M Intestinal epithelial cells secrete exosome-like vesicles Gastroenterology 2001 121 337 349 10.1053/gast.2001.26263 11487543

[B40] Hunter MP Ismail N Zhang X Aguda BD Lee EJ Yu L Xiao T Schafer J Lee ML Schmittgen TD Detection of microRNA expression in human peripheral blood microvesicles PLoS ONE 2008 3 e3694 10.1371/journal.pone.0003694 19002258 PMC2577891

[B41] Kosaka N Iguchi H Yoshioka Y Takeshita F Matsuki Y Ochiya T Secretory Mechanisms and Intercellular Transfer of MicroRNAs in Living Cells J Biol Chem 2010 285 17442 17452 10.1074/jbc.M110.107821 20353945 PMC2878508

[B42] Taylor DD Doellgast GJ Quantitation of peroxidase-antibody binding to membrane fragments using column chromatography Anal Biochem 1979 98 53 59 10.1016/0003-2697(79)90704-8 396817

[B43] Rabinowits G Gercel-Taylor C Day JM Taylor DD Kloecker GH Exosomal microRNA: a diagnostic marker for lung cancer Clin Lung Cancer 2009 10 42 46 10.3816/CLC.2009.n.006 19289371

[B44] Skog J Wurdinger T van Rijn S Meijer DH Gainche L Sena-Esteves M Curry WT Jr Carter BS Krichevsky AM Breakefield XO Glioblastoma microvesicles transport RNA and proteins that promote tumour growth and provide diagnostic biomarkers Nat Cell Biol 2008 10 1470 1476 10.1038/ncb1800 19011622 PMC3423894

[B45] Valenti R Huber V Iero M Filipazzi P Parmiani G Rivoltini L Tumor-released microvesicles as vehicles of immunosuppression Cancer Res 2007 67 2912 2915 10.1158/0008-5472.CAN-07-0520 17409393

[B46] Baj-Krzyworzeka M Szatanek R Weglarczyk K Baran J Zembala M Tumour-derived microvesicles modulate biological activity of human monocytes Immunol Lett 2007 113 76 82 10.1016/j.imlet.2007.07.014 17825925

[B47] Cortez MA Calin GA MicroRNA identification in plasma and serum: a new tool to diagnose and monitor diseases Expert Opin Biol Ther 2009 9 703 711 10.1517/14712590902932889 19426115

[B48] Chim SS Shing TK Hung EC Leung TY Lau TK Chiu RW Lo YM Detection and characterization of placental microRNAs in maternal plasma Clin Chem 2008 54 482 490 10.1373/clinchem.2007.097972 18218722

[B49] Luo SS Ishibashi O Ishikawa G Ishikawa T Katayama A Mishima T Takizawa T Shigihara T Goto T Izumi A Human villous trophoblasts express and secrete placenta-specific microRNAs into maternal circulation via exosomes Biol Reprod 2009 81 717 729 10.1095/biolreprod.108.075481 19494253

[B50] Wang GK Zhu JQ Zhang JT Li Q Li Y He J Qin YW Jing Q Circulating microRNA: a novel potential biomarker for early diagnosis of acute myocardial infarction in humans Eur Heart J 2010 31 659 666 20159880 10.1093/eurheartj/ehq013

[B51] Adachi T Nakanishi M Otsuka Y Nishimura K Hirokawa G Goto Y Nonogi H Iwai N Plasma MicroRNA 499 as a Biomarker of Acute Myocardial Infarction Clin Chem 2010 56 1183 1185 10.1373/clinchem.2010.144121 20395621

[B52] Wang K Zhang S Marzolf B Troisch P Brightman A Hu Z Hood LE Galas DJ Circulating microRNAs, potential biomarkers for drug-induced liver injury Proc Natl Acad Sci USA 2009 106 4402 4407 10.1073/pnas.0813371106 19246379 PMC2657429

[B53] Wang JF Yu ML Yu G Bian JJ Deng XM Wan XJ Zhu KM Serum miR-146a and miR-223 as potential new biomarkers for sepsis Biochem Biophys Res Commun 2010 394 184 188 10.1016/j.bbrc.2010.02.145 20188071

[B54] Vasilescu C Rossi S Shimizu M Tudor S Veronese A Ferracin M Nicoloso MS Barbarotto E Popa M Stanciulea O MicroRNA fingerprints identify miR-150 as a plasma prognostic marker in patients with sepsis PLoS One 2009 4 e7405 10.1371/journal.pone.0007405 19823581 PMC2756627

[B55] Murata K Yoshitomi H Tanida S Ishikawa M Nishitani K Ito H Nakamura T Plasma and synovial fluid microRNAs as potential biomarkers of rheumatoid arthritis and osteoarthritis Arthritis Res Ther 2010 12 R86 10.1186/ar3013 20470394 PMC2911870

[B56] Tanaka M Oikawa K Takanashi M Kudo M Ohyashiki J Ohyashiki K Kuroda M Down-regulation of miR-92 in human plasma is a novel marker for acute leukemia patients PLoS ONE 2009 4 e5532 10.1371/journal.pone.0005532 19440243 PMC2678255

[B57] Ng EK Chong WW Jin H Lam EK Shin VY Yu J Poon TC Ng SS Sung JJ Differential expression of microRNAs in plasma of patients with colorectal cancer: a potential marker for colorectal cancer screening Gut 2009 58 1375 1381 10.1136/gut.2008.167817 19201770

[B58] Schetter AJ Harris CC Plasma microRNAs: a potential biomarker for colorectal cancer? Gut 2009 58 1318 1319 10.1136/gut.2009.176875 19749133

[B59] He L Thomson JM Hemann MT Hernando-Monge E Mu D Goodson S Powers S Cordon-Cardo C Lowe SW Hannon GJ Hammond SM A microRNA polycistron as a potential human oncogene Nature 2005 435 828 833 10.1038/nature03552 15944707 PMC4599349

[B60] Huang Z Huang D Ni S Peng Z Sheng W Du X Plasma microRNAs are promising novel biomarkers for early detection of colorectal cancer Int J Cancer 2010 127 118 126 19876917 10.1002/ijc.25007

[B61] Wang J Chen J Chang P LeBlanc A Li D Abbruzzesse JL Frazier ML Killary AM Sen S MicroRNAs in plasma of pancreatic ductal adenocarcinoma patients as novel blood-based biomarkers of disease Cancer Prev Res (Phila Pa) 2009 2 807 813 10.1158/1940-6207.CAPR-09-0094 PMC5859193 19723895

[B62] Frankel LB Christoffersen NR Jacobsen A Lindow M Krogh A Lund AH Programmed cell death 4 (PDCD4) is an important functional target of the microRNA miR-21 in breast cancer cells J Biol Chem 2008 283 1026 1033 10.1074/jbc.M707224200 17991735

[B63] Lou Y Yang X Wang F Cui Z Huang Y MicroRNA-21 promotes the cell proliferation, invasion and migration abilities in ovarian epithelial carcinomas through inhibiting the expression of PTEN protein Int J Mol Med 2010 26 819 827 10.3892/ijmm_00000530 21042775

[B64] Gironella M Seux M Xie MJ Cano C Tomasini R Gommeaux J Garcia S Nowak J Yeung ML Jeang KT Tumor protein 53-induced nuclear protein 1 expression is repressed by miR-155, and its restoration inhibits pancreatic tumor development Proc Natl Acad Sci USA 2007 104 16170 16175 10.1073/pnas.0703942104 17911264 PMC2042180

[B65] Szafranska AE Davison TS John J Cannon T Sipos B Maghnouj A Labourier E Hahn SA MicroRNA expression alterations are linked to tumorigenesis and non-neoplastic processes in pancreatic ductal adenocarcinoma Oncogene 2007 26 4442 4452 10.1038/sj.onc.1210228 17237814

[B66] Crosby ME Kulshreshtha R Ivan M Glazer PM MicroRNA regulation of DNA repair gene expression in hypoxic stress Cancer Res 2009 69 1221 1229 10.1158/0008-5472.CAN-08-2516 19141645 PMC2997438

[B67] Resnick KE Alder H Hagan JP Richardson DL Croce CM Cohn DE The detection of differentially expressed microRNAs from the serum of ovarian cancer patients using a novel real-time PCR platform Gynecol Oncol 2009 112 55 59 10.1016/j.ygyno.2008.08.036 18954897

[B68] Lodes MJ Caraballo M Suciu D Munro S Kumar A Anderson B Detection of cancer with serum miRNAs on an oligonucleotide microarray PLoS One 2009 4 e6229 10.1371/journal.pone.0006229 19597549 PMC2704963

[B69] Yu SL Chen HY Chang GC Chen CY Chen HW Singh S Cheng CL Yu CJ Lee YC Chen HS MicroRNA signature predicts survival and relapse in lung cancer Cancer Cell 2008 13 48 57 10.1016/j.ccr.2007.12.008 18167339

[B70] Schetter AJ Leung SY Sohn JJ Zanetti KA Bowman ED Yanaihara N Yuen ST Chan TL Kwong DL Au GK MicroRNA expression profiles associated with prognosis and therapeutic outcome in colon adenocarcinoma JAMA 2008 299 425 436 10.1001/jama.299.4.425 18230780 PMC2614237

[B71] Calin GA Ferracin M Cimmino A Di Leva G Shimizu M Wojcik SE Iorio MV Visone R Sever NI Fabbri M A MicroRNA signature associated with prognosis and progression in chronic lymphocytic leukemia N Engl J Med 2005 353 1793 1801 10.1056/NEJMoa050995 16251535

[B72] Brase JC Johannes M Schlomm T Falth M Haese A Steuber T Beissbarth T Kuner R Sultmann H Circulating miRNAs are correlated with tumor progression in prostate cancer Int J Cancer 2010 Epub 20473869 10.1002/ijc.25376

[B73] Heneghan HM Miller N Lowery AJ Sweeney KJ Newell J Kerin MJ Circulating microRNAs as novel minimally invasive biomarkers for breast cancer Ann Surg 2010 251 499 505 10.1097/SLA.0b013e3181cc939f 20134314

[B74] Hu Z Chen X Zhao Y Tian T Jin G Shu Y Chen Y Xu L Zen K Zhang C Shen H Serum microRNA signatures identified in a genome-wide serum microRNA expression profiling predict survival of non-small-cell lung cancer J Clin Oncol 2010 28 1721 1726 10.1200/JCO.2009.24.9342 20194856

[B75] Nasser MW Datta J Nuovo G Kutay H Motiwala T Majumder S Wang B Suster S Jacob ST Ghoshal K Down-regulation of micro-RNA-1 (miR-1) in lung cancer. Suppression of tumorigenic property of lung cancer cells and their sensitization to doxorubicin-induced apoptosis by miR-1 J Biol Chem 2008 283 33394 33405 10.1074/jbc.M804788200 18818206 PMC2586284

[B76] Heneghan HM Miller N Kerin MJ Circulating miRNA Signatures: Promising Prognostic Tools for Cancer J Clin Oncol 2010 28 e573 574 10.1200/JCO.2010.29.8901 20697097

[B77] Chen C Ridzon DA Broomer AJ Zhou Z Lee DH Nguyen JT Barbisin M Xu NL Mahuvakar VR Andersen MR Real-time quantification of microRNAs by stem-loop RT-PCR Nucleic Acids Res 2005 33 e179 10.1093/nar/gni178 16314309 PMC1292995

[B78] Lusi EA Passamano M Guarascio P Scarpa A Schiavo L Innovative electrochemical approach for an early detection of microRNAs Anal Chem 2009 81 2819 2822 10.1021/ac8026788 19331434

[B79] Kozomara A Griffiths-Jones S miRBase: integrating microRNA annotation and deep-sequencing data Nucleic Acids Res 2010 Epub 21037258 10.1093/nar/gkq1027 PMC3013655

[B80] Liu CJ Kao SY Tu HF Tsai MM Chang KW Lin SC Increase of microRNA miR-31 level in plasma could be a potential marker of oral cancer Oral Dis 2010 16 360 364 10.1111/j.1601-0825.2009.01646.x 20233326

[B81] Wong TS Liu XB Wong BY Ng RW Yuen AP Wei WI Mature miR-184 as Potential Oncogenic microRNA of Squamous Cell Carcinoma of Tongue Clin Cancer Res 2008 14 2588 2592 10.1158/1078-0432.CCR-07-0666 18451220

[B82] Liang Y Ridzon D Wong L Chen C Characterization of microRNA expression profiles in normal human tissues BMC Genomics 2007 8 166 10.1186/1471-2164-8-166 17565689 PMC1904203

[B83] Zhu W Qin W Atasoy U Sauter ER Circulating microRNAs in breast cancer and healthy subjects BMC Res Notes 2009 2 89 10.1186/1756-0500-2-89 19454029 PMC2694820

[B84] Kroh EM Parkin RK Mitchell PS Tewari M Analysis of circulating microRNA biomarkers in plasma and serum using quantitative reverse transcription-PCR (qRT-PCR) Methods 2010 50 298 301 10.1016/j.ymeth.2010.01.032 20146939 PMC4186708

[B85] Tsujiura M Ichikawa D Komatsu S Shiozaki A Takeshita H Kosuga T Konishi H Morimura R Deguchi K Fujiwara H Circulating microRNAs in plasma of patients with gastric cancers Br J Cancer 2010 102 1174 1179 10.1038/sj.bjc.6605608 20234369 PMC2853097

[B86] Rao X Di Leva G Li M Fang F Devlin C Hartman-Frey C Burow ME Ivan M Croce CM Nephew KP MicroRNA-221/222 confers breast cancer fulvestrant resistance by regulating multiple signaling pathways Oncogene 2010 Epub 21057537 10.1038/onc.2010.487 PMC3342929

